# Loss of glutamate signaling from the thalamus to dorsal striatum impairs motor function and slows the execution of learned behaviors

**DOI:** 10.1038/s41531-018-0060-6

**Published:** 2018-08-02

**Authors:** Erica J. Melief, Jonathan W. McKinley, Jonathan Y. Lam, Nicole M. Whiteley, Alec W. Gibson, John F. Neumaier, Charles W. Henschen, Richard D. Palmiter, Nigel S. Bamford, Martin Darvas

**Affiliations:** 1 Department of Pathology, University of Washington, Seattle, WA USA; 2 Departments of Pediatrics, Neurology and Cellular and Molecular Physiology, Yale University, New Haven, CT USA; 3 Psychology Program, Seattle University, Seattle, WA USA; 4 Graduate Program in Neuroscience, University of Washington, Seattle, WA USA; 5 Department of Psychiatry and Behavioral Sciences, University of Washington, Seattle, WA USA; 6 Department of Pharmacology, University of Washington, Seattle, WA USA; 7 Department of Biochemistry, University of Washington, Seattle, WA USA; 8 Howard Hughes Medical Institute, University of Washington, Seattle, WA USA; 9 Department of Neurology, University of Washington, Seattle, WA USA

## Abstract

Parkinson’s disease (PD) is primarily associated with the degeneration of midbrain dopamine neurons, but it is now appreciated that pathological processes like Lewy-body inclusions and cell loss affect several other brain regions, including the central lateral (CL) and centromedian/parafascicular (CM/PF) thalamic regions. These thalamic glutamatergic neurons provide a non-cortical excitatory input to the dorsal striatum, a major projection field of dopamine neurons. To determine how thalamostriatal signaling may contribute to cognitive and motor abnormalities found in PD, we used a viral vector approach to generate mice with loss of thalamostriatal glutamate signaling specifically restricted to the dorsal striatum (CAV2^Cre^-*Slc17a6*^*lox/lox*^ mice). We measured motor function and behaviors corresponding to cognitive domains (visuospatial function, attention, executive function, and working memory) affected in PD. CAV2^Cre^-*Slc17a6*^*lox/lox*^ mice were impaired in motor coordination tasks such as the rotarod and beam-walk tests compared with controls (CAV2^Cre^-*Slc17a6*^*+/+*^ mice). They did not demonstrate much cognitive impairment in the Morris water maze or a water U-maze, but had slower processing reaction times in those tests and in a two-way active avoidance task. These mice could model an aspect of bradyphrenia, the slowness of thought that is often seen in patients with PD and other neurological disorders.

## Brain connectivity: Interrupting cognitive processing speed

Mice in which glutamate signaling from the thalamus to dorsal striatum has been genetically inactivated mimic the slowness of thought that is often observed in patients with Parkinson’s disease (PD). The midbrain and striatum are the brain regions that are most affected in PD, however, it is increasingly recognized that cell loss in other areas of the brain also contribute to disease symptoms. Martin Darvas at the University of Washington, Seattle, USA, and colleagues found that disrupting the excitatory input from thalamic projection neurons into the dorsal striatum affected motor coordination and balance in mice. Although these mice did not have significant impairments in spatial learning and memory, they were slower at reacting to cues and executing learned behaviors suggesting that they could be used to test new approaches for treating this specific cognitive symptom of PD.

## Introduction

PD is a well-known neurodegenerative disease whose hallmark symptoms include postural instability, bradykinesia, resting tremor, and dementia. A principal characteristic pathology of PD is the loss of midbrain dopamine-producing neurons that project to the dorsal striatum and the presence of α-synuclein-containing Lewy body (LB) inclusions in the midbrain.^1^ While the presence of LBs in the midbrain is a primary pathology in PD, it is now recognized that LBs and aggregated α-synuclein can be found in many other brain structures, and that these may contribute to the symptoms and signs of this disease. Brain regions that have recently come under scrutiny in the pathology of PD are the intra-laminar midline nuclei of the thalamus. The intra-laminar nuclei consist of the anterior central lateral (CL) nucleus, the center-median (CM) nucleus, and the parafascicular (PF) nucleus.^2,3^ LB inclusions are demonstrated in the CL of PD patients along with neuronal loss in the more posterior CM and PF (CM/PF) complex.^4^ Diffusion tensor magnetic resonance imaging (MRI) in patients with PD shows that disruption of thalamic projections is present during the initial stages of disease,^5^ and other MRI studies show alterations in the overall shape of the thalamus as early as ~4 years after the initial clinical diagnosis that reflect presumed volume loss in the CL, CM, and PF.^6^ In addition to these structural changes, functional MRI data demonstrate changed thalamic activity in early stages of PD,^7^ in which loss of function lead to poor executive function and memory.^8–10^

In contrast to cortical afferents that express the vesicular glutamate transporter 1 (VGLUT1), projections from CL/CM/PF neurons express VGLUT2. Thalamostriatal projections provide the main non-cortical glutamatergic innervation of the striatum, and they share major targets with dopaminergic projections: spiny projection neurons (SPNs) and cholinergic interneurons,^2^ both of which have been implicated in mechanisms of learning and memory and in cognitive impairment in models of neurodegeneration.^11–14^ Most thalamostriatal projections originate from intra-laminar thalamic nuclei, including neurons in the CL, CM, and PF nuclei.^15^ Given the convergence of thalamic glutamatergic and midbrain dopaminergic signaling in the dorsal striatum and their relevance for PD, we asked whether disruption of thalamostriatal projections would affect motor and/or cognitive function in the absence of dopamine neuron loss. To date, there have been few direct investigations of how CL and CM/PF nuclei can regulate abnormal movements and cognition, yet evidence from human imaging studies and the presence of pathology in PD suggest these thalamic nuclei play a critical role in the development of clinical disease.

To investigate how this understudied brain region might alter cognition, we used transgenic mice with conditional *Slc17a6* alleles (which encode the vesicular glutamate transporter, VGLUT2) and injected a Cre-expressing canine adenovirus (CAV2-Cre) into the dorsal striatum (DS). CAV2-Cre is retrogradely transported to the cell bodies of thalamic projection neurons whose terminals reside in the striatal injection field. This strategy inactivates VGLUT2 selectively in neurons that project axon terminals to the DS without lesioning the thalamus. Using this approach, we specifically disable thalamostriatal glutamatergic signaling in the DS without altering thalamic output to other brain regions or triggering loss of cofactors that would be inherent in neuronal loss. These animals were tested using behavior tests that measure motor and cognitive domains that are affected in PD.

## Results

### Reduction of slc17a6 expression in CL and CM/PF

**Fig. 1:**
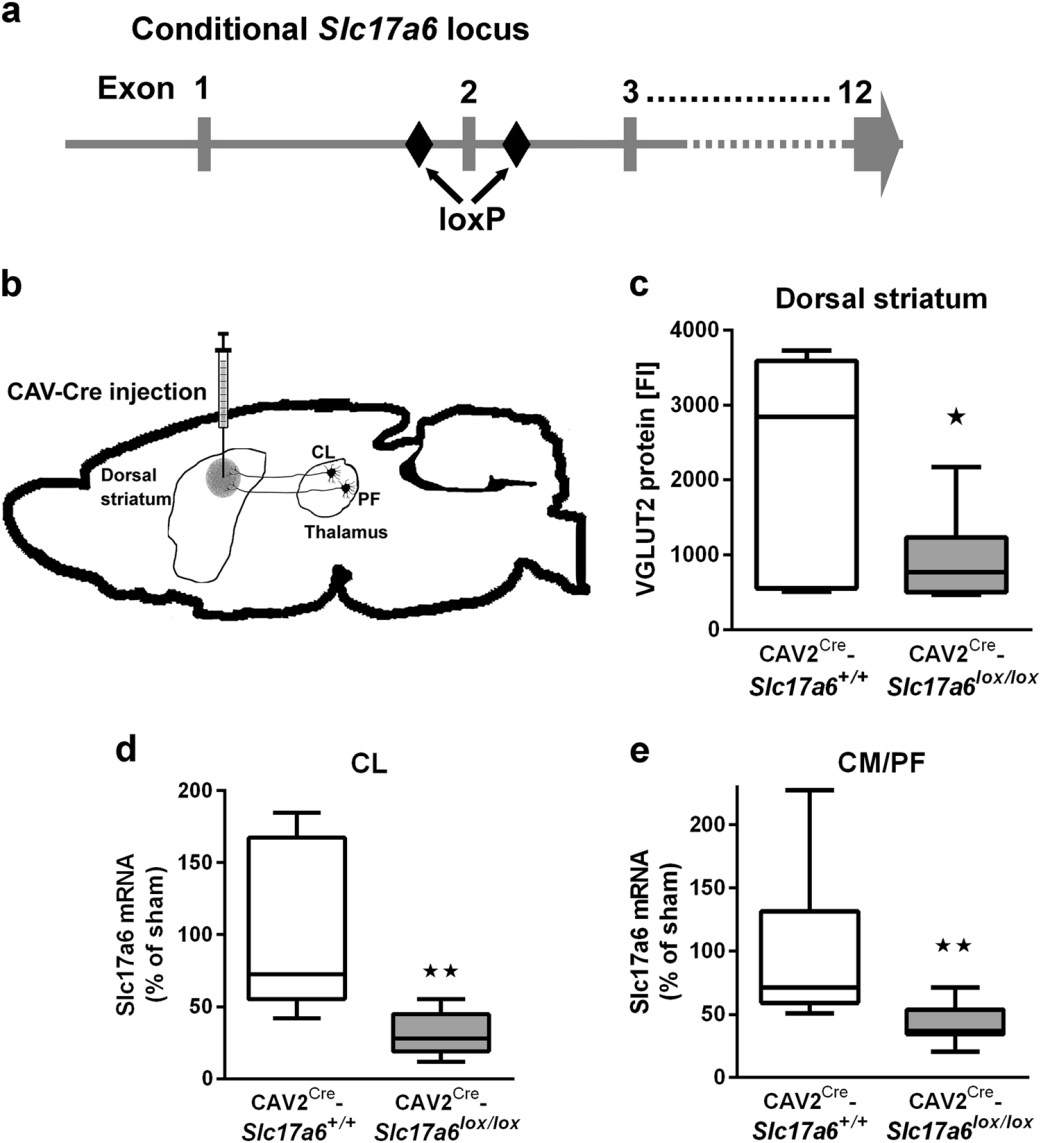
*Ablation of VGlut2 mRNA and protein following CAV-Cre injection into the dorsal striatum in CAV2*^*Cre*^*-Slc17a6*^*lox/lox*^ *mice*. **a** Schematic representation showing the position of loxP sites inserted into the Slc17a6 locus of Slc17a6^lox/lox^ mice. **b** Illustration of needle placement for CAV2-Cre injections into CAV2^Cre^-*Slc17a6*^*+/+*^ controls and CAV2^Cre^-*Slc17a6*^*lox/lox*^ mice. **c** Expression of VGLUT2 protein in the dorsal striatum of CAV2^Cre^-*Slc17a6*^*+/+*^ controls (*n* = 6) and CAV2^Cre^-*Slc17a6*^*lox/lox*^ (*n* = 8) mice as measured by LUMINEX quantification. **d** Expression of *Slc17a6* mRNA in the CL thalamic nucleus of CAV2^Cre^-*Slc17a6*^*+/+*^ controls (*n* = 10) and CAV2^Cre^-*Slc17a6*^*lox/lox*^ (*n* = 12) mice. **e** Expression of Slc17a6 mRNA in the CM/PF thalamic nuclei of CAV2^Cre^-*Slc17a6*^*+/+*^ controls (*n* = 10) and CAV2^Cre^-*Slc17a6*^*lox/lox*^ (*n* = 12) mice. Data in all figures are shown as mean ± standard error of mean (SEM). Statistical significance of pairwise comparison of means from CAV2^Cre^-*Slc17a6*^*+/+*^ controls and CAV2^Cre^-*Slc17a6*^*lox/lox*^ mice are shown. *p < 0.05

Figure 1a shows a schematic of the *Slc17a6* gene with loxP sites flanking exon 2, while Fig. 1b is a diagram of the basic strategy for inactivation of VGLUT2 in neurons that project to the dorsal striatum by retrograde CAV2-Cre transport. To determine the effectiveness of VGLUT2 inactivation via CAV2-Cre injection in the dorsal striatum, we first analyzed the expression of VGLUT2 protein in tissue punches taken from the dorsal striatum (Fig. 1c) using a Luminex assay with magnetic beads coupled to an antibody that recognizes VGLUT2. Next, we analyzed expression of *Slc17a6* mRNA in tissue punches taken from the CL (Fig. 1d) and CM/PF (Fig. 1e) nuclei of the thalamus using real time PCR (RT-PCR). CAV2-Cre injection into *Slc17a6*^*lox/lox*^ mice (CAV2^Cre^-*Slc17a6*^*lox/lox*^) resulted in a ~40% reduction of VGLUT2 protein in the dorsal striatum (*p < 0.05), and in a ~40% reduction of *Slc17a6* mRNA expression in the CL and CM/PF (both **p < 0.01); all percentages were normalized to expression levels in *Slc17a6*^*+/+*^ mice that were also injected with CAV2-Cre (CAV2^Cre^-*Slc17a6*^*+/+*^). *Slc17a6* mRNA expression data was normalized by *Rn18s* expression in all analyses. We note that we would not expect 100% loss because the thalamic tissue punches include VGLUT2-positive neurons that project to brain regions other than the dorsal striatum. To assess the extent of expression and location of CAV2-Cre injections, we injected CAV2-Cre into a cohort of *Rosa26*^*fstdTomato*^ mice,^16^ using the same injection procedure and coordinates as for *Slc17a6*^*lox/lox*^ and *Slc17a6*^*+/+*^ mice. This cohort was not used for behavior or electrophysiology experiments and was used solely to estimate the spread of CAV2-Cre after injection into the striatum. CAV2-Cre injection into *Rosa26*^*fstdTomato*^ mice resulted in tdTomato expression in the injected area in the dorsal striatum that extended to striatal regions ~0.5 mm anterior and ~0.5 mm posterior of the targeted region (Supplemental Fig. S1).

### Thalamostriatal excitatory postsynaptic currents are eliminated in striatal projection neurons from CAV2^Cre^-Slc17a6^lox/lox^ mice

**Fig. 2:**
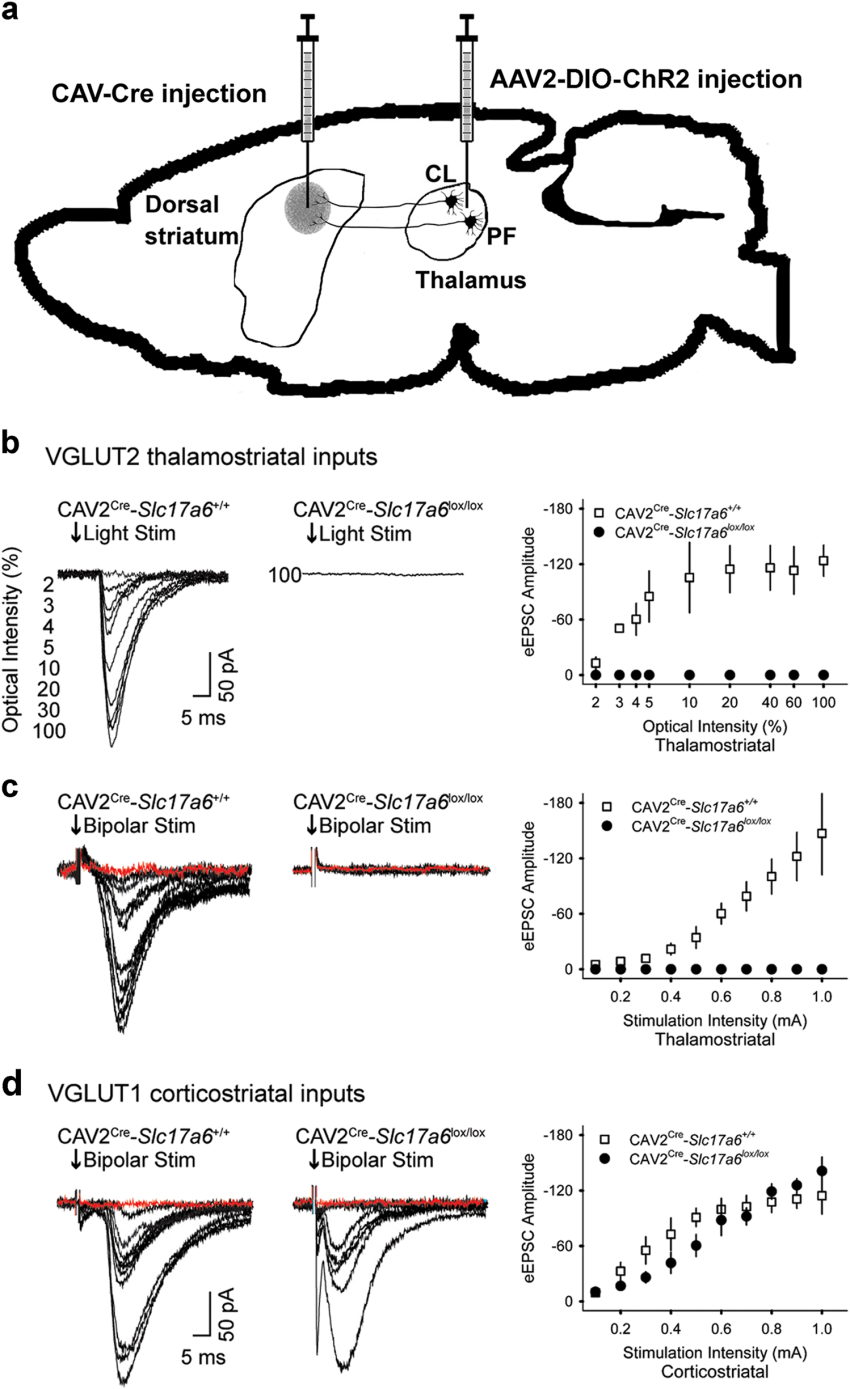
*Electrophysiology on VGlut1 and VGlut2 striatal afferents*. **a** Illustration in the sagittal plane demonstrates the placement of CAV2-Cre and AAV2-ChR2 injections into CAV2^Cre^-*Slc17a6*^*+/+*^ controls and CAV2^Cre^-*Slc17a6*^*lox/lox*^ mice. **b** Representative traces show that light activation of VGLUT2 thalamostriatal synapses produces opsin-generated eEPSCs in SPNs from CAV2^Cre^-*Slc17a6*^*+/+*^ controls *n* = 5; left) but not in CAV2^Cre^-*Slc17a6*^*lox/lox*^ mice (*n* = 9; center). Amplitude of light activated eEPSCs in SPNs from CAV2^Cre^-*Slc17a6*^*+/+*^ controls and CAV2^Cre^-*Slc17a6*^*lox/lox*^ mice (right). **c** Bipolar stimulation of thalamostriatal projections produced eEPSCs in SPNs from CAV2^Cre^-*Slc17a6*^*+/+*^ controls (*n* = 5; left) but not in CAV2^Cre^-*Slc17a6*^*lox/lox*^ mice (*n* = 4; center). Amplitude of bipolar activated eEPSCs in SPNs from CAV2^Cre^-*Slc17a6*^*+/+*^ controls and CAV2^Cre^-*Slc17a6*^*lox/lox*^ mice (right). For **c** and **d**, synaptic currents were eliminated after adding NBQX to the bath solution, indicating that they arose from activation of glutamatergic receptors (red traces). **d** Bipolar stimulation of corticostriatal projections produced eEPSCs in SPNs from both CAV2^Cre^-*Slc17a6*^*+/+*^ controls (*n* = 5; left) and CAV2^Cre^-*Slc17a6*^*lox/lox*^ mice (*n* = 5; center). Amplitude of bipolar activated eEPSCs in SPNs from CAV2^Cre^-*Slc17a6*^*+/+*^ controls and CAV2^Cre^-*Slc17a6*^*lox/lox*^ mice (right). Data in all figures are shown as mean ± standard error of mean (SEM)

We used electrophysiological experiments in striatal SPNs to functionally validate that CAV2-Cre mediated loss of VGLUT2 in thalamic neurons disrupted the synaptic release of glutamate from thalamostriatal terminals. We used an optogenetic approach with CAV2^Cre^-*Slc17a6*^*lox/lox*^ mice and CAV2^Cre^-*Slc17a6*^*+/+*^ mice to confirm that we had selectively targeted VGLUT2 expression in thalamostriatal projections from the CL and CM/PF to SPNs. For this purpose, *Slc17a6*^*lox/lox*^ and *Slc17a6*^*+/+*^ mice received a striatal injection of CAV2-Cre, which results in the excision of the *Slc17a6* gene and elimination of VGLUT2 expression in thalamostriatal projection neurons in the *Slc17a6*^*lox/lox*^ mice. To ensure the target neurons were thalamic in origin, we made additional injections of AAV2-EF1α-DIO-hChR2(H134R)-mCherry (AAV2-ChR2) in the right and left CL/CM/PF nuclei of the thalamus to express channel rhodopsin in neurons originating in these regions (Fig. 2a). Together, this ensures that thalamostriatal neurons will produce evoked EPSCs in response to light, and that elimination of VGLUT2 from these neurons will abolish that response. After 4 weeks, mice were sacrificed for whole-cell electrophysiology in brain slices and evoked excitatory postsynaptic currents (eEPSCs) were measured in striatal SPNs (Fig. 2b–d). The mean membrane capacitance (121.2 ± 6 and 134 ± 10.5 pF), input resistance (98.3 ± 5.2 and 100.6 ± 12.5 MΩ), and time constants (1.6 ± 0.1 and 1.8 ± 0.1 ms) were similar in SPNs from CAV2^Cre^-*Slc17a6*^*lox/lox*^ mice and CAV2^Cre^-*Slc17a6*^*+/+*^ mice. Opsin activation by blue light of thalamic afferents in the striatum produced eEPSCs in SPNs (*n* = 5) from AAV2-ChR2 injected CAV2^Cre^-*Slc17a6*^*+/+*^ mice, but not in AAV2-ChR2 injected CAV2^Cre^-*Slc17a6*^*lox/lox*^ mice (*n* = 9 cells; Fig. 2b). This suggested a reduction in glutamate release from thalamostriatal afferents in CAV2^Cre^-*Slc17a6*^*lox/lox*^ mice. As a positive control, we electrically stimulated thalamostriatal inputs using bipolar electrodes. Similar to the optogenetic experiments, stimulation (0.1–1 mA) of thalamic afferents with bipolar electrodes produced eEPSCs in striatal SPNs (*n* = 5) of CAV2^Cre^-*Slc17a6*^*+/+*^ mice, but not in CAV2^Cre^-*Slc17a6*^*lox/lox*^ mice (*n* = 4 cells; Fig. 2c). In contrast, as a positive control and to ensure the specificity of the thalamostriatal targeting, bipolar stimulation of VGLUT1 corticostriatal inputs produced eEPSCs in CAV2^Cre^-*Slc17a6*^*+/+*^ and CAV2^Cre^-*Slc17a6*^*lox/lox*^ mice (*n* = 5 cells; Fig. 2d).

### Motor deficits

**Fig. 3:**
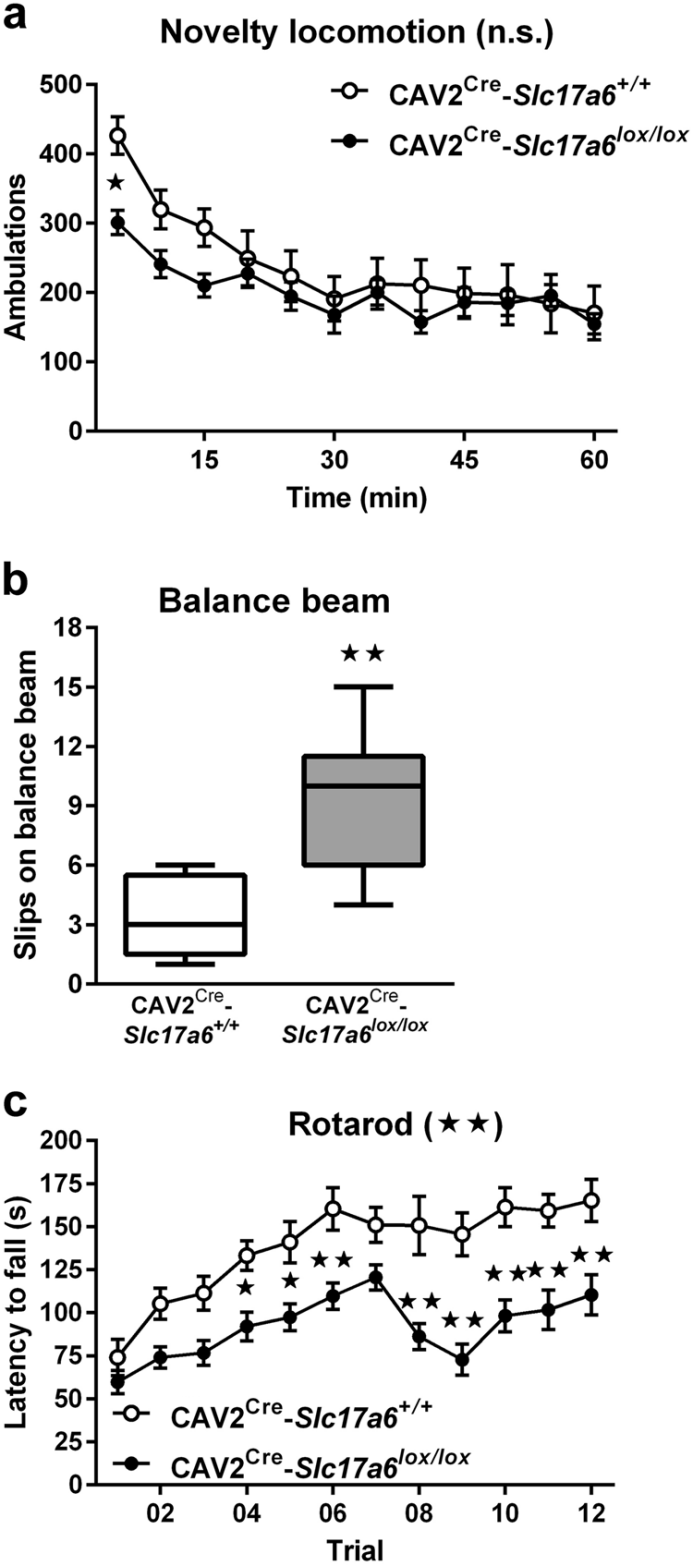
*Motor behavioral tasks*. **a** Spontaneous locomotion in response to a novel environment by CAV2^Cre^-*Slc17a6*^*+/+*^ controls (*n* = 9) and CAV2^Cre^-*Slc17a6*^*lox/lox*^ (*n* = 8) mice. **b** Motor coordination on a balance beam by CAV2^Cre^-*Slc17a6*^*+/+*^ controls (*n* = 5) and CAV2^Cre^-*Slc17a6*^*lox/lox*^ (*n* = 9) mice. **c** Motor-skill learning on the rotating rotarod by CAV2^Cre^-*Slc17a6*^*+/+*^ controls (*n* = 11) and CAV2^Cre^-*Slc17a6*^*lox/lox*^ (*n* = 17 mice. Data are shown as mean ± SEM. Statistical significance of genotype effects (repeated-measures two-way ANOVA) are shown in the headings of panels a and c (not significant, n.s.). Statistical significance of pairwise comparison of means from CAV2^Cre^-*Slc17a6*^*+/+*^ controls and CAV2^Cre^-*Slc17a6*^*lox/lox*^ mice are shown. *p < 0.05 and **p < 0.01

Because of the role of the dorsal striatum on locomotion, we assessed the effects of removing glutamatergic input from the thalamus to the dorsal striatum on motor behaviors in CAV2^Cre^-*Slc17a6*^*lox/lox*^ mice. Although there was no significant difference in spontaneous novelty-induced locomotion (Fig. 3a), CAV2^Cre^-*Slc17a6*^*lox/lox*^ mice had significantly more slips on the balance beam task (Fig. 3b) and performed significantly worse over time on the rotarod apparatus (Fig. 3c) as compared with CAV2^Cre^-*Slc17a6*^*+/+*^ controls (ANOVA, *p < 0.05, **p < 0.01), indicating an impairment in learned motor behaviors and innate balance.

### Spatial learning

**Fig. 4:**
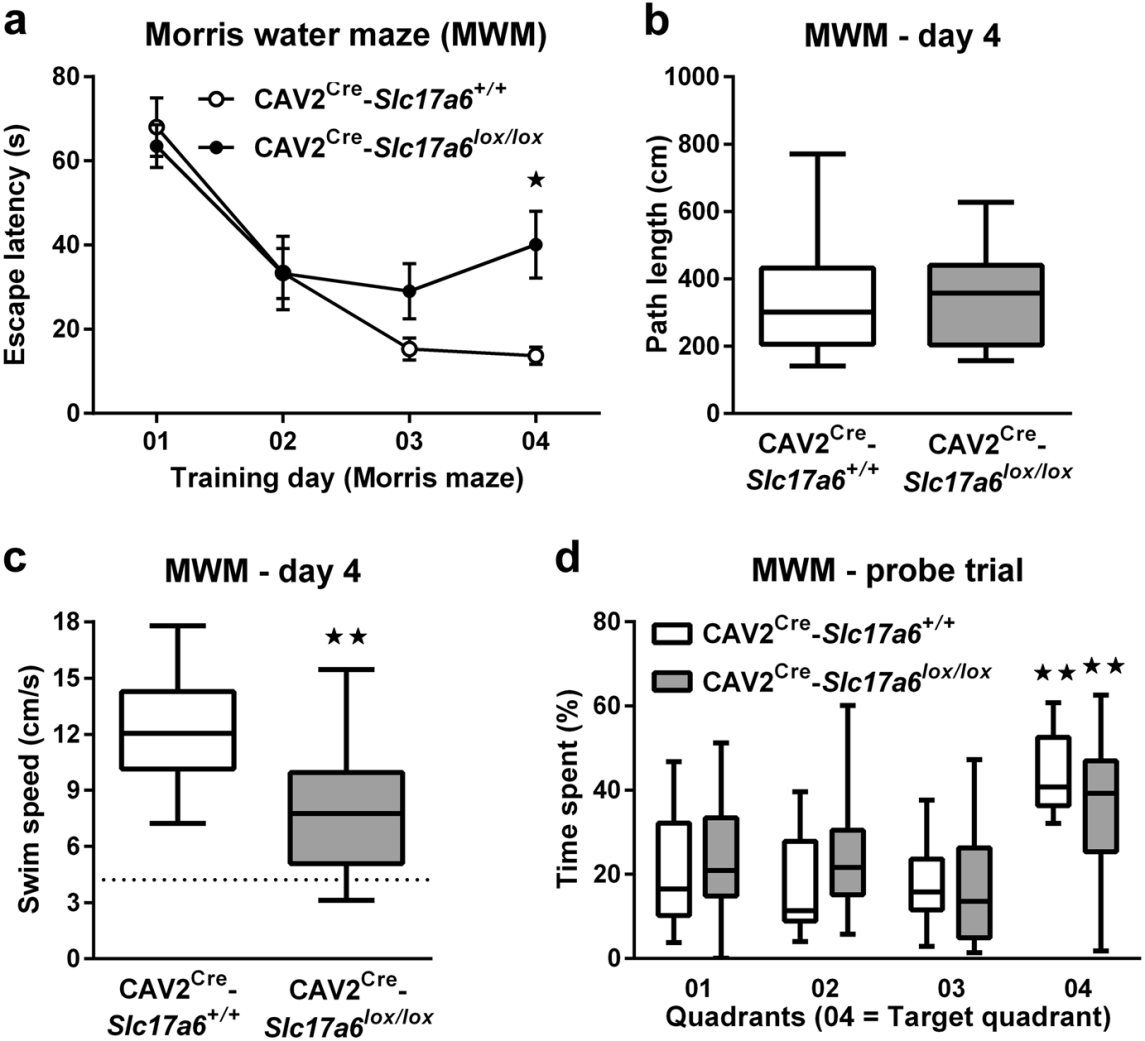
*Morris water maze behavioral task*. **a** Latency (s) to climb onto a submerged platform in the Morris water maze, **b** path length (cm), **c** swim speed (cm/s), and **d** percentage time spent in the quadrants of the maze by CAV2^Cre^-*Slc17a6*^*+/+*^ controls (*n* = 11) and CAV2^Cre^-*Slc17a6*^*lox/lox*^ (*n* = 17) mice. Data are shown as mean ± SEM. Statistical significance of pairwise comparison of means from CAV2^Cre^-*Slc17a6*^*+/+*^ controls and CAV2^Cre^-*Slc17a6*^*lox/lox*^ mice are shown in **a** and **c**. Statistical significance of preference for quadrant 4 is shown in **d**. *p < 0.05 and **p < 0.01

Among the non-motor effects of PD, cognitive impairment is one of the most detrimental to a patient’s functionality. Behavioral tasks of learning and memory are commonly used to assess cognitive impairment models of PD and dopaminergic dysfunction, and the dorsal striatum has been implicated in these tasks.^17,18^ To assess the effect of reduced thalamostriatal glutamate signaling on spatial learning and memory, we tested animals in a Morris water maze. There was no overall significant effect of genotype on latency to find the escape platform (Fig. 4a), despite a significant increase in the latency of CAV2^Cre^-*Slc17a6*^*lox/lox*^ animals to escape on day 4 (ANOVA, *p < 0.05). To determine the cause of the increased escape latency on day 4, we analyzed animals’ path length (Fig. 4b) and swim speed (Fig. 4c); path length did not differ from CAV2^Cre^-*Slc17a6*^*+/+*^ controls, but swim speed was significantly reduced in CAV2^Cre^-*Slc17a6*^*lox/lox*^ animals (ANOVA, **p < 0.05), indicating one mechanism for the increased escape latency on day 4. However, their swim speed was higher than would be expected by a simple division of path length by latency to escape (Fig. 4c dashed line), indicating an impairment in continuity of swimming rather than a simple motor impairment.

To evaluate spatial memory, we tested animals in a probe trial 24 h after the last training day (Fig. 4d). All animals spent the most time in quadrant 4 (the previous location of the escape platform), indicating retention of the escape location. There was no significant difference between experimental and control mice.

**Fig. 5:**
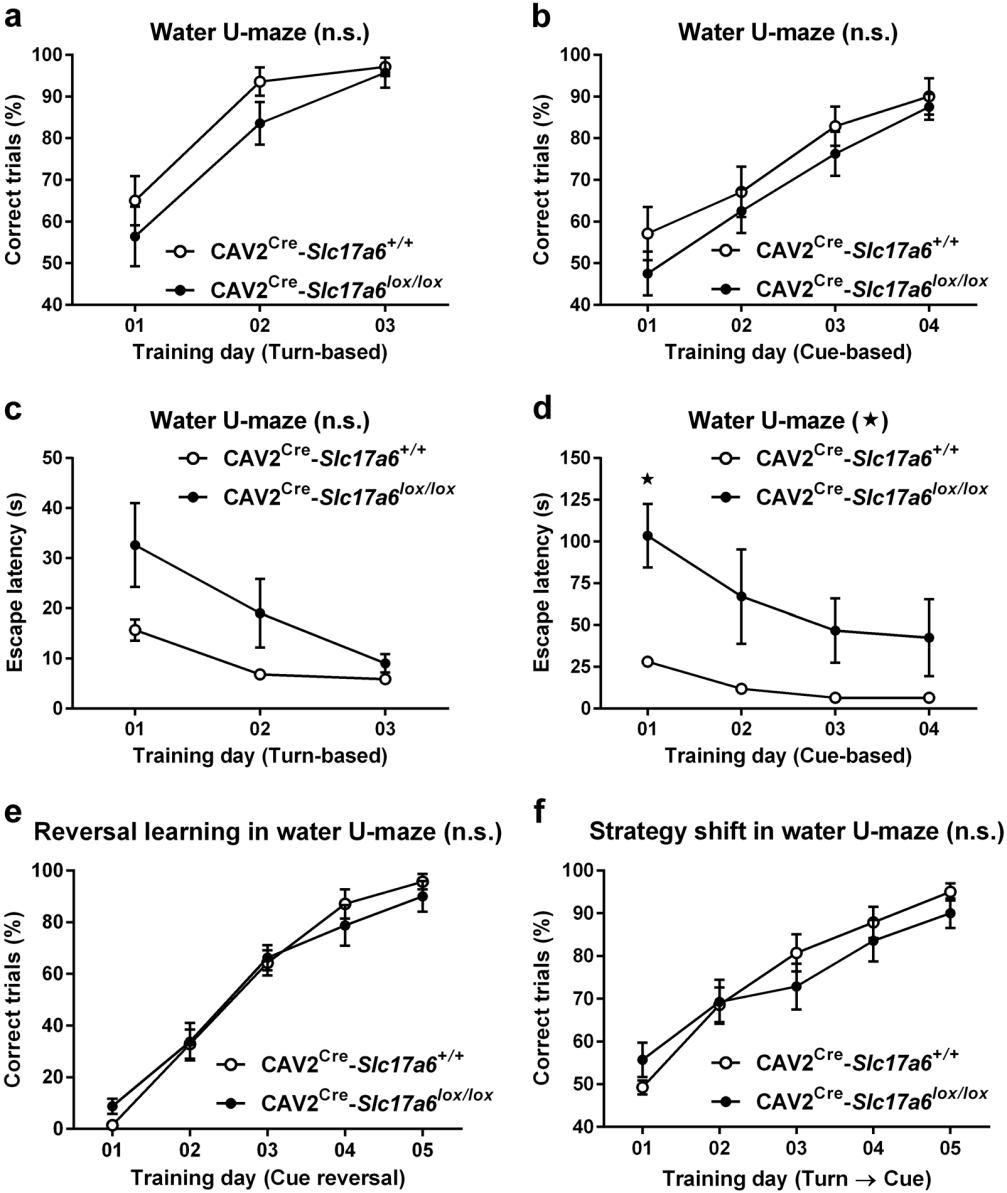
*Water U-maze behavioral task*. **a** Percentage of correct trials during turn-dependent (CAV2^Cre^-*Slc17a6*^*+/+*^ controls *n* = 14, and CAV2^Cre^-*Slc17a6*^*lox/lox*^ mice *n* = 14) and **b** cue-dependent (CAV2^Cre^-*Slc17a6*^*+/+*^ controls *n* = 7 and CAV2^Cre^-*Slc17a6*^*lox/lox*^ mice *n* = 8) water-escape learning in the water-U maze. **c** Latency (s) to climb onto the platform during turn-dependent (CAV2^Cre^-*Slc17a6*^*+/+*^ controls *n* = 14, and CAV2^Cre^-*Slc17a6*^*lox/lox*^ mice *n* = 14) and **d** cue-dependent (CAV2^Cre^-*Slc17a6*^*+/+*^ controls *n* = 7, and CAV2^Cre^-*Slc17a6*^*lox/lox*^ mice *n* = 8) water-escape learning in the water-U maze. **e** Percentage of correct trials during reversal-learning (cue-reversal, CAV2^Cre^-*Slc17a6*^*+/+*^ controls *n* = 7, and CAV2^Cre^-*Slc17a6*^*lox/lox*^ mice *n* = 8) and **f** strategy-shift learning (switch from turn-based escape strategy to cue-based strategy, CAV2^Cre^-*Slc17a6*^*+/+*^ controls *n* = 14, and CAV2^Cre^-*Slc17a6*^*lox/lox*^ mice *n* = 14) in the water-U maze. Data are shown as mean ± SEM. Statistical significance of genotype effects (repeated-measures two-way ANOVA) are shown in the headings of each panel. Statistical significance of pairwise comparison of means from CAV2^Cre^-*Slc17a6*^*+/+*^ controls and CAV2^Cre^-*Slc17a6*^*lox/lox*^ mice are shown in panel d. *p < 0.05

To determine the effect of VGLUT2 loss on learning and memory in a test independent of swim speed, we tested animals in the water U-maze, which has been shown to be sensitive to striatal lesion and dopamine impairment.^17,18^ Both CAV2^Cre^-*Slc17a6*^*lox/lox*^ mice and CAV2^Cre^-*Slc17a6*^*+/+*^ controls were able to learn the location of an escape platform using a turn-based escape protocol (Fig. 5a) and a cue-based escape protocol (Fig. 5b) with no significant differences between groups in number of correct turns made per day. Interestingly, however, the latency to reach the escape platform in the cue-based escape protocol was significantly higher for CAV2^Cre^-*Slc17a6*^*lox/lox*^ than for CAV2^Cre^-*Slc17a6*^*+/+*^ controls (Fig. 5d) (ANOVA, *p < 0.05). The trend was not significant in the turn-based escape protocol (Fig. 5c), ruling out motor impairment as a cause for the increased latency in the cue-based escape protocol.

### Cognitive flexibility

To assess cognitive flexibility, a higher executive function that has also been shown to be disrupted in animals with PD-like lesions,^17^ mice that had been trained to find the escape platform using a cue-based escape protocol were then challenged by switching the cue for the escape (for example, animals that had been trained to use the black-cue as the escape were then switched to the white side). Both CAV2^Cre^-*Slc17a6*^*lox/lox*^ mice and CAV2^Cre^-*Slc17a6*^*+/+*^ controls were able to learn the cue-reversal equally well (Fig. 5e). Likewise, animals that had been trained to find the escape platform using a turn-based protocol were then challenged to switch to a cue-based protocol. Again, both CAV2^Cre^-*Slc17a6*^*lox/lox*^ mice and CAV2^Cre^-*Slc17a6*^*+/+*^ controls were equally able to adapt to the strategy shift and find the escape platform (Fig. 5f), indicating no difference in overall learning and memory or cognitive flexibility.

### Active avoidance

**Fig. 6:**
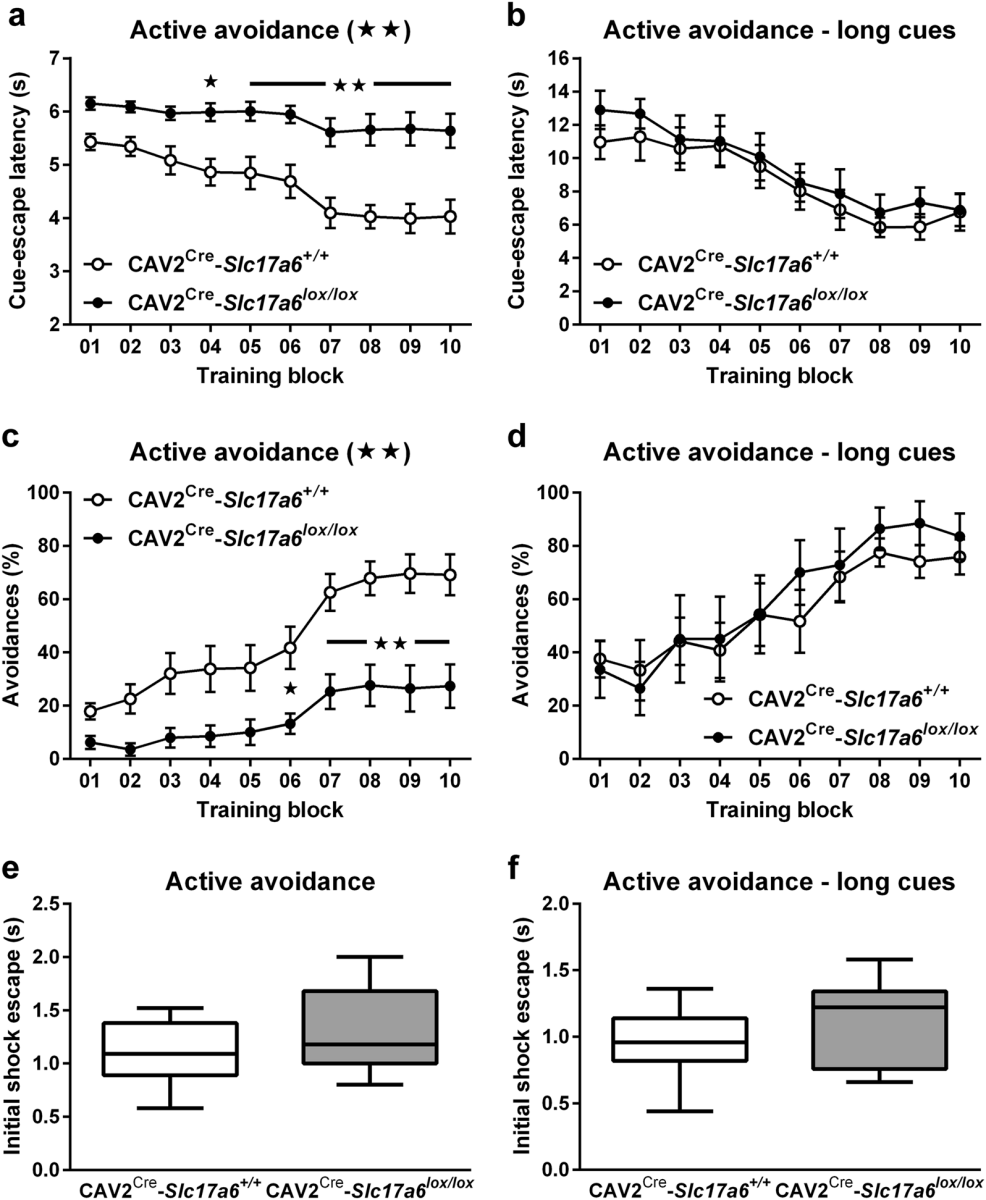
*Active avoidance behavioral task*. **a** Cue-escape latencies (s) from 5-s (CAV2^Cre^-*Slc17a6*^*+/+*^ controls *n* = 12, and CAV2^Cre^-*Slc17a6*^*lox/lox*^ mice *n* = 17) and from **b** 14-s auditory cues (CAV2^Cre^-*Slc17a6*^*+/+*^ controls *n* = 7, and CAV2^Cre^-*Slc17a6*^*lox/lox*^ mice *n* = 7). **c** Percentage of active avoidance responses to 5-s (CAV2^Cre^-*Slc17a6*^*+/+*^ controls *n* = 12, and CAV2^Cre^-*Slc17a6*^*lox/lox*^ mice *n* = 17) and from **d** 14-s auditory cues (CAV2^Cre^-*Slc17a6*^*+/+*^ controls *n* = 7, and CAV2^Cre^-*Slc17a6*^*lox/lox*^ mice *n* = 7). **e** Shock-escape latencies (s) for first ten shocks following 5-s (CAV2^Cre^-*Slc17a6*^*+/+*^ controls *n* = 12, and CAV2^Cre^-*Slc17a6*^*lox/lox*^ mice *n* = 17) and **f** 14-s auditory cues (CAV2^Cre^-*Slc17a6*^*+/+*^ controls *n* = 7, and CAV2^Cre^-*Slc17a6*^*lox/lox*^ mice *n* = 7). Data are shown as mean ± SEM. Statistical significance of genotype effects (repeated-measures two-way ANOVA) are shown in the headings of each panel. Statistical significance of pairwise comparison of means from CAV2^Cre^-*Slc17a6*^*+/+*^ controls and CAV2^Cre^-*Slc17a6*^*lox/lox*^ mice are shown in **a**, **c**, and **e**. *p < 0.05 and **p < 0.01

To further assess animals’ learning and memory in a non-motor dependent test, we used the two-way active avoidance paradigm, a fear based learning and memory task which has been shown to be sensitive to dopaminergic neurotoxins and responsive to Parkinson’s therapies and provides a behavioral learning and memory test which is not dependent on swim speed.^19^ Animals were trained that a 5-s auditory cue predicted a foot shock that could be avoided by escaping into the other chamber of the testing box before the cue ended. CAV2^Cre^-*Slc17a6*^*+/+*^ controls decreased their escape latency (Fig. 6a) and increased the number of successful “avoidances” over time (Fig. 6c), while CAV2^Cre^-*Slc17a6*^*lox/lox*^ animals performed significantly worse than controls overall (ANOVA, *p < 0.05, **p < 0.01), indicating impaired learning and memory. To rule out inherent differences in response to the shock, we measured the reaction time to the first ten foot shocks on day 1 (Fig. 6e). There was no significant difference between the two groups in initial response to the foot shocks. Because of the increased latency to escape and initiate movement seen in the different water mazes, we hypothesized that these animals might require a longer time to respond to the cue. To determine the effect of cue-length on escape, we then repeated the experiments with a 14-s (instead of 5 s) auditory cue. When presented the longer cue, CAV2^Cre^-*Slc17a6*^*lox/lox*^ animals performed similarly to CAV2^Cre^-*Slc17a6*^*+/+*^ controls in latency to escape (Fig. 6b) and number of successful avoidances (Fig. 6d). As was observed with the shorter cue, there was no significant difference between groups in their initial response to the first ten foot shocks on day 1 (Fig. 6f).

## Discussion

Cognitive impairment reported in PD patients often includes deficits in executive function (cognitive flexibility) and visuospatia l function.^20–25^ Lewy bodies, the neuropathological hall mark of PD and PD-like disorders, can be observed in the thalamus in later stages of the disease,^4^ and imaging studies in healthy volunteers and in patients with thalamic strokes provide evidence for a role of the CM/PF nucleus in attentional processes,^8,26–29^ cognitive flexibility and recognition memory.^9,10,30^ In this study we sought to determine whether specific loss of VGLUT2 in neurons projecting to the dorsal striatum would produce cognitive and motor deficits similar to those seen in PD and in patients with thalamic stroke in the absence of a neuronal lesion. To address this, we used a genetic approach to selectively cause permanent loss of VGLUT2 expression in neurons projecting to the dorsal striatum. Our approach targeted both CL and CM/PF *Slc17a6* mRNA expression. While previous studies have shown that Lewy bodies are restricted to cells in the anterior intralaminar thalamic nuclei, it has also been shown that there is neuronal loss in the posterior thalamic nuclei in the absence of α−synuclein aggregation.^4,31,32^ By targeting both the anterior and posterior nuclei using our retrograde CAV2-Cre viral approach, we were able to examine the effect of signal loss from both of these populations in a manner unattainable from other genetic or toxin-induced approaches.

We found that CAV2^Cre^-*Slc17a6*^*lox/lox*^ animals showed significant motor deficits relative to CAV2^Cre^-*Slc17a6*^*+/+*^ controls that were comparable to established toxin models of PD.^33^ CAV2^Cre^-*Slc17a6*^*lox/lox*^ animals had significantly more slips on the beam walk apparatus and a significantly decreased latency to fall in the rotarod than controls (CAV2^Cre^-*Slc17a6*^*+/+*^ mice). Interestingly, CAV2^Cre^-*Slc17a6*^*lox/lox*^ animals were not significantly different from controls in novelty-induced locomotion, with the exception of the first 5 min of the trial. This perhaps indicates a delay in processing the novelty of the environment, which would depend on intact cognitive processing. However, CAV2^Cre^-*Slc17a6*^*lox/lox*^ showed relatively normal spatial learning and cognitive flexibility compared with CAV2^Cre^-*Slc17a6*^*+/+*^ controls. All animals were able to learn the location of the escape platform in the Morris water maze with similar path lengths and similar time spent in the correct quadrant in a probe trial, indicating no overall deficit in spatial learning and memory. Interestingly, however, the CAV2^Cre^-*Slc17a6*^*lox/lox*^ mice were slightly slower than CAV2^Cre^-*Slc17a6*^*+/+*^ controls at finding the escape platform on day 4. As the path length to the platform was the same for both groups, we analyzed swim speed and found that CAV2^Cre^-*Slc17a6*^*lox/lox*^ animals had an active swim speed that was faster than would be expected by a simple length/time equation (Fig. 5c, dashed line). This indicates that CAV2^Cre^-*Slc17a6*^*lox/lox*^ mice were impaired in the *continuity* of their swimming, either in starting the swim or by repeated stop-start effects. Likewise, CAV2^Cre^-*Slc17a6*^*lox/lox*^ animals demonstrated intact ability to learn a cue-based or turn-based rule for locating the escape platform in the water U-maze, as measured by the number of correct choices the animal made each day. However, CAV2^Cre^-*Slc17a6*^*lox/lox*^ animals had a significantly increased latency to escape in the cue-based rule paradigm than CAV2^Cre^-*Slc17a6*^*+/+*^ controls (the trend for the turn-based escape rule was not significant). Together, these data indicate that, while CAV2^Cre^-*Slc17a6*^*lox/lox*^ mice are capable of learning an escape paradigm, they are literally “slower” at executing that escape than CAV2^Cre^-*Slc17a6*^*+/+*^ controls. To further investigate this interesting observation, we tested CAV2^Cre^-*Slc17a6*^*lox/lox*^ mice using a two-way active avoidance learning paradigm. Here we found that while CAV2^Cre^-*Slc17a6*^*lox/lox*^ animals have similar response times to a foot shock as CAV2^Cre^-*Slc17a6*^*+/+*^ controls, they were unable to avoid the shock when the cue time was 5 s. However, when we increased the cue time to 14 s, CAV2^Cre^-*Slc17a6*^*lox/lox*^ animals were comparable to CAV2^Cre^-*Slc17a6*^*+/+*^ controls in their escape latency and shock avoidance learning. This indicates that the CAV2^Cre^-*Slc17a6*^*lox/lox*^ animals required a longer time to processes the cue and its indication of an impending shock. Together with the slower execution of the water maze escape, we believe that CAV2^Cre^-*Slc17a6*^*lox/lox*^ mice may be a model of bradyphrenia, the “slowness of thought” which is a symptom of cognitive impairment found in PD.

While CAV2^Cre^-*Slc17a6*^*lox/lox*^ mice had no learning deficit in our cognitive flexibility task, others have shown that transient pharmacological inactivation of the PF in rats produced a deficit in behavioral flexibility.^12^ This difference could be due to the specific experimental setup used in our study, which allowed animals to self-correct after they made a wrong decision and which did not include a cutoff time-out. Another possibility is that while we only inactivated VGLUT2-mediated neurotransmission, local pharmacological inhibition in the PF may have inactivated additional cell types in the PF that could contribute to deficits in behavioral flexibility. Pharmacological inhibition might also affect other transmitters/modulators produced by thalamic neurons. Finally, although most PF neurons are thought to project to the striatum, some thalamic PF neurons also project to other brain regions, including cortical areas that may also be involved in cognitive flexibility. Interestingly, another study that used an intersectional approach to selectively ablate thalamostriatal neurons in the PF did not observe any motor deficits, but rather impairments of attention processes and visual discrimination in a reaction-time task.^34^ We believe that our observations of CAV2^Cre^-*Slc17a6*^*lox/lox*^ mice provide evidence that inactivation of glutamatergic signaling from the CL, or perhaps both CL and CM/PF, is most likely necessary for motor impairment, which imitates bradykinesia in PD. It is possible that the bradyphrenia-like behavior that we observed in various cognitive behaviors also produces impairment in rotarod learning. We suggest that the bradyphrenia-like behavior observed in CAV2^Cre^-*Slc17a6*^*lox/lox*^ mice would most likely result in impairments in the reaction-time task employed by Kato et al, which required animals to respond within a 5-s time window.^34^ In fact, our two-way active avoidance data provide clear support for this interpretation. At this time, it is unclear whether the CL has a specific contribution to cognitive behaviors and future experiments are needed to address this question.

Taken together, our data clearly confirm an involvement of thalamostriatal glutamatergic CL and CM/PF neurons in bradykinesia and bradyphrenia-like behaviors in an animal model, and thereby implicate the loss of cell function in these nuclei as contributors to cognitive and motor impairment in PD. It is not clear whether the mechanism of this contribution may also occur through glutamatergic action on dopaminergic neurons or cholinergic interneurons (ChIs), which generate high levels of acetylcholine in the striatum and which have been implicated in cognitive behaviors.^35,36^ Previous studies have shown that thalamostriatal innervation of ChIs play a critical role in certain types of learning and memory.^11,14^ It is likely that a reduction in thalamostriatal excitability would promote changes in burst firing and pauses of these tonically-active ChIs, which may modify acetylcholine availability and participate in the behavioral phenotype. Together with our current data, we believe that this important observation may explain some of the clinical limitations, especially with regard to cognition, of drugs which primarily act on dopamine receptors.^37–39^ Indeed, there is currently great interest in establishing novel non-dopaminergic therapeutic approaches for PD and our CAV2^Cre^-*Slc17a6*^*lox/lox*^ mice are a suitable model to test new approaches. One important future approach will be to combine use of CAV2^Cre^-*Slc17a6*^*lox/lox*^ mice with other animal models used in PD research and determine the effects of complex PD pathologies in multiple brain regions on physiology and behavior.

## Methods

### Animals

All procedures were performed in accordance with the United States Public Health Service Guide for Care and Use of Laboratory Animals and were approved by the Institutional Animal Care and Use Committee at the University of Washington and Yale University. Heterozygous *Slc17a6*^*+/lox*^ mice on a C57BL/6 background have exon 2 flanked by LoxP sites^40^; they were bred to generate *Slc17a6*^*+/+*^ wild-type controls and *Slc17a6*^*lox/lox*^ littermates. Mice of both sexes at the age of 3 to 5 months old were used for all experiments. No sex differences were observed in any of the tests. All mice were housed in groups of 3–5 animals under a 12-hr, light-dark cycle (6AM-6PM) in a temperature-controlled environment with food and water available ad libitum. In the absence of intervention, *Slc17a6*^*lox/lox*^ mice appear phenotypically normal compared with wild- type littermates. To assess the extent and location of CAV2-Cre injections, we injected CAV2-Cre into a cohort of *Rosa26*^*fstdTomato*^ mice,^16^ using the same injection procedure and coordinates as for *Slc17a6*^*lox/lox*^ and *Slc17a6*^*+/+*^ mice.

### Reagents and antibodies

RNA extraction kits (Ambion) and primers (Applied Biosystems) were purchased from Thermo Scientific (Waltham, MA). The iScript cDNA synthesis kit was purchased from Bio-Rad (Hercules, CA). DNase I was purchased from Worthington Biochemical (Lakewood, NJ). Magnetic MagPlex beads and bead conjugation kits (Bio-Plex Amine Coupling kit) were purchased from Bio-Rad (Hercules, CA). Streptavidin-phycoerythrin (SA-PE) was purchased from Bio-Rad (Hercules, CA). The following primary antibodies were used: monoclonal anti-VGLUT2 (clone EPR21085) from Abcam (Cambridge, MA), biotinylated anti-VGLUT2 from Lifespan Biosciences (Seattle, WA), monoclonal anti α-tubulin (DM1A) from Cell Signaling Technologies (Danvers, MA).

### CAV2-cre and AAV-opsin injections

CAV2-Cre (0.5 μl, 3 × 10^12^ viral genomes per μl) was stereotaxically injected bilaterally into the dorsal striatum (0.5 mm anterior to Bregma, ± 1.75 mm lateral to midline, 3.0 mm ventral from the skull surface) using the Allen Mouse Brain Atlas as a guide.^41^ All animals used for behavioral experiments received striatal CAV2-Cre injection: CAV2-Cre injected *Slc17a6*^*+/+*^ animals were used as controls (CAV2^Cre^-*Slc17a6*^*+/+*^ controls) and CAV2-Cre injected *Slc17a6*^*lox/lox*^ littermates were used to generate CAV2^Cre^-*Slc17a6*^*lox/lox*^ mice.

For electrophysiological experiments, CAV2^Cre^-*Slc17a6*^*+/+*^ controls and CAV2^Cre^-*Slc17a6*^*lox/lox*^ littermates also received stereotaxic injections of the opsin AAV2-EF1α-DIO-hChR2(H134R)-mCherry (0.5 μl AAV2-ChR2; 3 × 10^9^ viral genomes per μl; UNC Vector Core) into the CL/CM/PF thalamic nuclei (−2.18 mm anterior to Bregma, ± 0.5 mm lateral to midline, 3.3 mm ventral from the skull surface).

During stereotaxic injections, animals were anesthetized with 1–2% isoflurane (Kent Scientific, Torrington, CT) and with lidocaine/bupivacaine (1.0 mg/kg and 0.5 mg/kg s.c.). After surgeries, analgesia was supplied by ketoprofen (5.0 mg/kg, s.c.). All animals were allowed to recover from surgeries for 4 weeks before they were submitted to further experimental procedures.

### Electrophysiology

eEPSCs were recorded in striatal SPNs obtained from CAV2^Cre^-*Slc17a6*^*+/+*^ controls and CAV2^Cre^-*Slc17a6*^*lox/lox*^ mice. Some mice received AAV2-EF1α-DIO-hChR2(H134R)-mCherry (0.5 µl; 3 × 10^9^ viral genomes/μl) injections into the CL/CM/PF (Bregma, *y*: +2.18, *x*: ±0.5, *z*:−3.3 mm). Standard techniques were used to prepare 250-µm slices.^42^ Experiments were performed using coronal (Bregma, +1.54 to 0.62 mm) or horizontal oblique brain striatal slice preparations that preserve corticostriatal and thalamostriatal connectivity.^43^ Mice were anesthetized with Beuthanasia (270 mg/kg i.p.) and decapitated. Brain slices were cut by vibratome in submerged in ice-cold, carbogenated (95% O_2_, 5% CO_2_) cutting solution containing (in mM): KCl (3), NaHCO_3_ (26), NaH_2_PO_4_ (1.25), MgSO_4_ (3.3), MgCl_2_ (1.7), CaCl_2_ (1), sucrose (236) and glucose (10) (pH 7.2–7.4, 290–310 mOsm). Slices were then transferred to an incubating chamber containing carbogenated artificial cerebrospinal fluid solution (aCSF) containing (in mM): NaCl (124), KCl (5), NaHCO_3_ (26), NaH_2_PO_4_ (1.25), MgCl_2_ (2), CaCl_2_ (2) and glucose (10) (pH 7.2–7.4, 290–310 mOsm) at 35 °C. After 1 h, slices were placed on the stage of an upright Olympus BX51WI microscope and submerged in continuously flowing carbogenated aCSF (3 ml/min) warmed to 35 °C.

Whole-cell patch clamp recordings in voltage clamp mode were obtained from SPNs, visualized in slices with the aid of florescence and infrared video microscopy coupled with differential interference contrast optics. SPNs were identified by size (8–12 µm), responses to current injection and basic membrane properties (input resistance, membrane capacitance and time constant).^44,45^ EPSCs were isolated by blocking gamma-aminobutyric acid (GABA_A_) receptors with bicuculline (10 µM). Cells were held at −70 mV to further minimize the contribution of GABA_A_-mediated events and that of voltage-gated conductances. Passive membrane properties were monitored throughout the recording and cells were removed from further analysis if the series resistance changed by >20%. The patch pipette (4–6 MΩ) contained the following internal solution (in mM): Cs-methanesulfonate (125), KCl (3), NaCl (4), MgCl_2_ (1), MgATP (5), EGTA (5), HEPES (8), Tris-GTP (1), Di-sodium phosphocreatine (10), leupeptin (0.1) and N-(2,6-dimethylphenylcarbamoylmethyl) triethylammonium bromide (4) (QX-314; pH 7.2–7.3, 270–280 mOsm). Cells were filled with Alexaflor 594 hydrazide (Invitrogen; 20 µM in internal solution). eEPSCs were evoked by either light stimulation (473 nm, 2-ms; 2–100%; CoolLED, Andover, UK) of the surrounding tissue or by electrical stimulation (0.1–1 mA) using twisted tungsten bipolar electrode (Plastics One, Roanoke, VA) applied every 30 s. Corticostriatal projections were stimulated by electrodes placed over cortical layers V–VI Thalamostriatal projections were stimulated by electrodes placed over the reticular nucleus (Supplemental Fig. S2). Stimulation intensity (was adjusted upwards in steps until a maximum peak amplitude was achieved without eliciting variable latencies or prolonged durations suggesting polysynaptic responses. At the conclusion of each experiment, the non- N-methyl-D-aspartic acid (NMDA) receptor antagonist sodium-2,3-dihydro-6-nitro-7-sulfamoyl-benzo[f]quinoxaline (2 µM; NBQX) was applied to confirm that currents were AMPA receptor mediated. Currents were Bessel filtered at 4 kHz and digitized at 50 µs using an IBM computer equipped with Digidata 1440 A data acquisition and pClamp10.2 software (Molecular Devices). Data was analyzed using Clampfit 10.6 software. Unless noted otherwise, drugs were obtained from either Sigma (St. Louis, MO) or Abcam Biochemicals (Cambridge, MA).

### VGLUT2 protein expression

To measure VGLUT2 protein expression, we collected tissue in 1-mm punches from the dorsal striatum. Tissue was immediately flash-frozen in liquid nitrogen and stored at −80 °C. Tissue was homogenized, followed by protein extraction. Total protein content was quantified for all samples using a standard BCA assay from Thermo Fisher Scientific (Waltham, MA). We first conducted a Western blotting confirmation experiment to determine a suitable antibody for the detection of VGLUT2 and then performed a Luminex assay to measure VGLUT2 levels.

#### Luminex

Monoclonal anti-VGLUT2 was conjugated to magnetic beads using the Bio-Plex Amine Coupling kit (Bio-Rad). For each animal we incubated 40 μg of total protein extract over night with 2500 MagPlex beads conjugated to anti-VGLUT2. Next, beads were collected using a strong magnetic plate, washed, and then incubated for 3.5 h with biotinylated anti-VGLUT2. Then, beads were collected and washed, and incubated for 1 h with SA-PE. Finally, beads were collected and washed, resuspended in phosphate buffered saline and the signals measured with a Bio-Plex 200 System from Bio-Rad (Hercules, CA). All Luminex reactions were performed as duplicates.

### Quantification of *Slc17a6* mRNA expression

To quantify expression of *Slc17a6* mRNA in the CL and CM/PF, we collected tissue in 1-mm punches from the CM and CM/PF nuclei of the thalamus. Tissue was immediately flash-frozen in liquid nitrogen and stored at −80 °C. Tissue was homogenized, followed by RNA extraction using Ambion PureLink®RNA kits, and then reverse-transcribed with iScript cDNA synthesis kits; we always used the same amount of RNA (100 ng) for cDNA synthesis. Primers for detection of *Slc17a6* and *Rn18s* were purchased from Applied Biosystems. RT-PCR was performed on an Applied Biosystems ViiA 7 Real-Time PCR System with the method of relative quantitation using normalization to 18 S ribosomal RNA (*Rn18s*) expression^46^; all PCR reactions were performed as triplicates and with the same amount of cDNA.

### Motor behavioral tests

#### Novelty locomotion

Locomotor activity was measured using static mouse cages (37.2 cm D × 23.4 cm W × 14 cm H) with 16 photo cells per side (Columbus Instruments) and an IBM computer to record beam breaks. Ambulations (two consecutive beam breaks) were measured in 5 min intervals over a 60-min test period in a novel mouse testing room.

#### Rotarod

Mice were tested for motor impairment in the rotarod test (Rotamex 4/8 system, Columbus Instruments) for three consecutive days. Mice received four trials per day with an intertrial interval (ITI) of 5–10 min. For each trial the rod accelerated from 4 to 40 rpm over the course of 5 min. Latency to fall was measured.

#### Beam walk

Mice were tested for innate motor balance and coordination in a beam walk test as previously described.^47^ Animals were placed on a 60-cm cylindrical rod (15 mm in diameter) that was elevated 30 cm above a cushioned table and the number of slips was recorded as they traversed the length of the rod. Mice that fell were placed back on the beam at the position where they fell and allowed to continue to the end.

### Learning behavioral tests

#### Morris water maze

*Spatial learning and memory* were measured using a modified version of the Morris water maze procedure as previously described.^47,48^ Mice were trained to locate a platform that was submerged in a circular pool (84-cm diameter) filled with opaque water using spatial cues provided outside the pool. Over a period of 4 days, animals received four training trials with an ITI of 3 to 5 min. Each trial ended if the animal reached the submerged platform within 90 s. If the animal failed to reach the platform within 90 s, the experimenter gently guided the animal to the platform. In either case, the animal was allowed to rest on top of the platform for 30 s. All trials were recorded with a camera and analyzed using Ethovision video-tracking software (Noldus, Wageningen, The Netherlands). Spatial learning was measured as the latency to reach the submerged platform and was averaged over the four trials of each training day. In addition, we used Ethovision software to calculate the average swim speed and path length of animals in the Morris maze. Following completion of spatial learning, spatial memory was measured using a 90 s probe trial (with the platform removed) 24 h following completion of the last training session. Video recordings of the probe trial were analyzed with Ethovision software and spatial memory was scored as the percentage of time spent in the quadrant of the pool where the platform was positioned during training.

#### Water U-maze

Ego-centric learning, cue-based learning, and cognitive flexibility were measured using in a water U-maze apparatus.^17,18^ Mice were released into a gray stem that ended in one black and one white arm choice. One arm always had an escape platform at the end which was not visible to the mice from the stem. The right-left orientation of the arms was alternated in an equal, pseudo-random manner. Mice were given ten trials per day to learn the location of the escape platform with an ITI of about 5 min. Trials lasted until the mouse found the escape platform or 5 min had elapsed, at which point the mouse was shown the location of the platform. The first 3 days of testing, mice were trained to find the escape platform using a directional, turn-based egocentric protocol (platform was always to the right or the left, regardless of arm color). Following this training, a cohort of mice were then challenged with a strategy-shift to a cue-based approach strategy, in which they were trained for 5 days to find the escape platform using a color-based approach (platform was always in the black or the white arm, regardless of direction). A separate cohort of animals were trained to find the escape platform using a cue-based protocol (platform was always in the black or the white arm, regardless of direction). A cohort of these mice was then trained in a reversal-learning strategy, in which the escape platform was switched to the other color cue. Percent correct trials per day and latency to escape were measured.

#### Active avoidance

Fear-based learning and response times were tested using a two-way active avoidance task, as previously described.^49^ Avoidance was assessed in an apparatus that consisted of two-chamber PACS-30 two-way shuttle boxes with an opaque wall containing an opening between the chambers (Columbus Instruments). The grid floor was made of stainless steel rods that were connected to a constant current shock generator that delivered a foot shock to the entire grid. Animal movements were detected by photo-beams in each chamber. A sound-cue generator was situated above the opening between the two compartments. Mice received 100 trials per day, each of which was followed by a 40-s ITI. Each trial started with the onset of a 5-s or 19-s sound cue (2.5 kHz, 80 dB). If the mouse moved to the other chamber within this time the sound cue was terminated. Avoidance failure was followed by the delivery of a 0.3-mA foot shock to the floor of the shuttle box (co-presented with the sound cue) that could last for a maximum of 2 s and was terminated if the mouse moved to the other chamber. Regardless of avoidance, the protocol resumed following the 40-s ITI, during which mice were allowed to shuttle freely between chambers. During each trial, the latency to escape from the sound cue, escape from the foot shock, and the number of avoidances during presentation of the sound cue were recorded. During each ITI only the number of shuttles between chambers was recorded. Results from each of the 20 trials or ITIs were combined into training a block.

### Statistics

Values given in the text and in the figures are indicated as mean ± SEM. Differences in mean values were assessed with Student’s *t*-tests or appropriate analyses of variance (ANOVAs) followed by multiple comparisons using Bonferroni *t*-tests. Statistical analyses were performed with Prism (GraphPad Prism, La Jolla, CA) and differences were considered significant if *p* < 0.05.

### Data availability

Authors can confirm that all relevant data are included in the paper and/ or its supplementary information Files.

## Electronic supplementary material

Supplementary material
