# Supplementary material for: Loss of glutamate signaling from the thalamus to dorsal striatum impairs motor function and slows the execution of learned behaviors
Source: NPJ Parkinsons Dis. 2018 Aug 2;4:23. doi: 10.1038/s41531-018-0060-6 (PMC6072777; doi:10.1038/s41531-018-0060-6)
Supplement: Supplementary file 1 — Supplementary material [file 41531_2018_60_MOESM1_ESM.pdf]

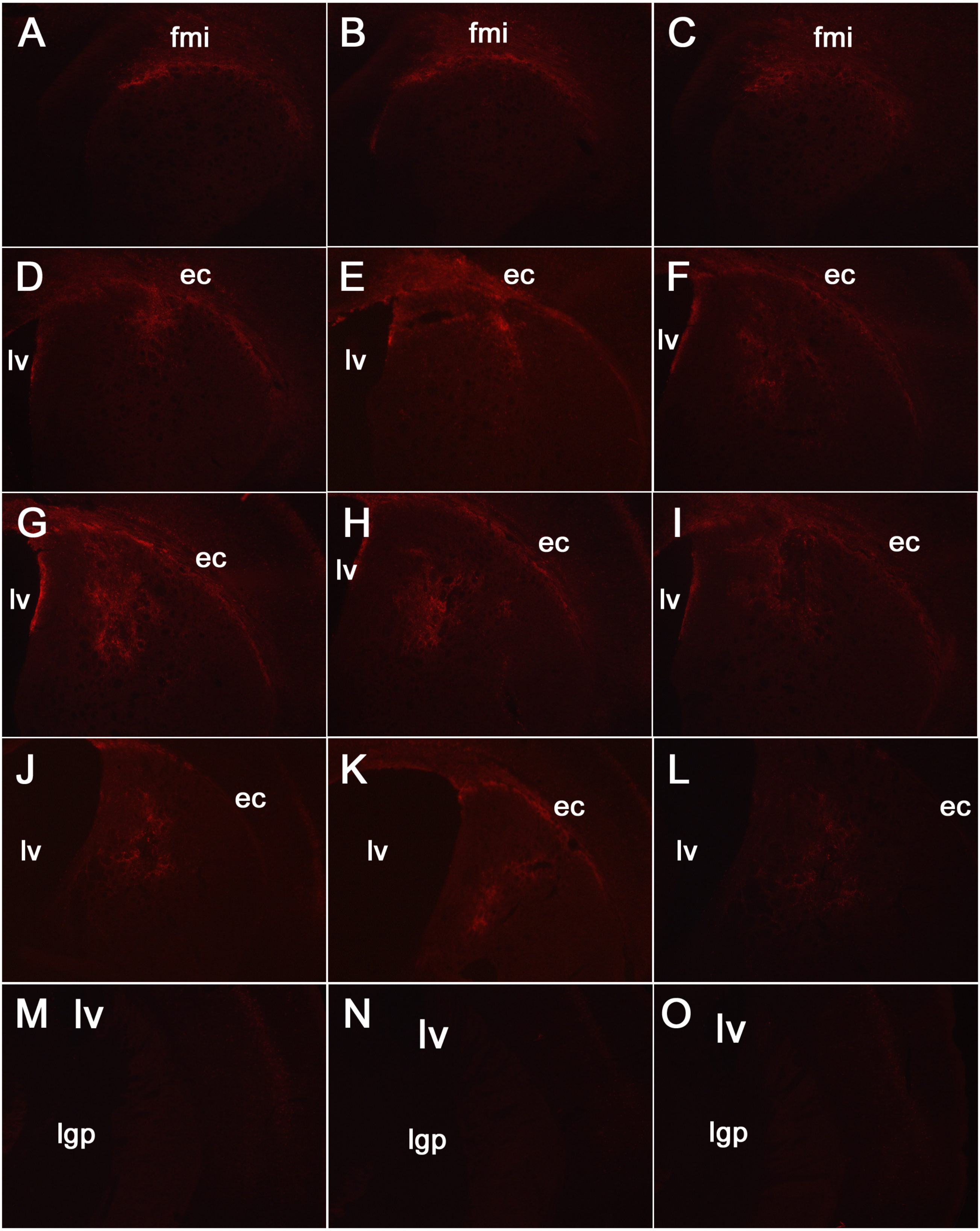
A
fmi
B
fmi
C
fmi
D
ec
lv
E
ec
lv
F
ec
lv
G
ec
lv
H
ec
lv
I
ec
lv
J
ec
lv
K
ec
lv
L
ec
lv
M
lv
lgp
N
lv
lgp
O
lv
lgp

Figure S1. *Coronal striatal tissue sections showing the typical location of area injected with CAV2-Cre in Rosa26^{fstdTomato} mice*. Virus-mediated activation of tdTomato expression is shown as red fluorescence. (A-C) Example sections at 1.5 mm anterior to Bregma from 3 different *Rosa26*^{fstdTomato} mice. (D-F) Example sections at 1.0 mm anterior to Bregma from 3 different *Rosa26*^{fstdTomato} mice. (G-I) Example sections at 0.5 mm anterior to Bregma from 3 different *Rosa26*^{fstdTomato} mice. (J-L) Example sections at 0.1 mm anterior to Bregma from 3 different *Rosa26*^{fstdTomato} mice. (M-O) Example sections at 0.5 mm posterior to Bregma from 3 different *Rosa26*^{fstdTomato} mice.

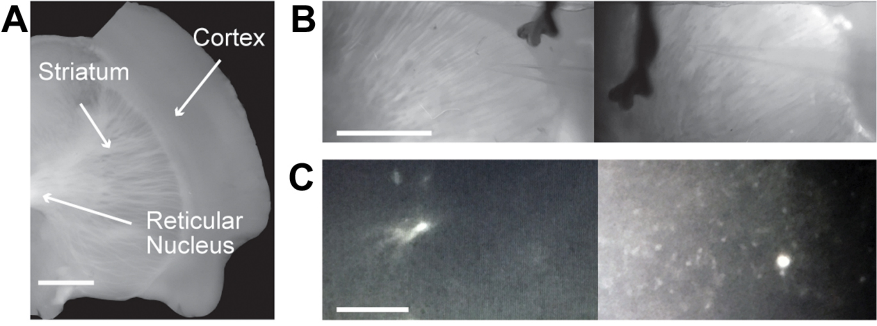
A
Cortex
Striatum
Reticular
Nucleus
B
C

Figure S2. *Placement of electrodes for stimulation and recording of VGlut1 and VGlut2 striatal afferents*. (A) Electrophysiology experiments were performed using horizontal oblique brain striatal slice preparations to preserve corticostriatal and thalamostriatal connectivity (Bar: 1 mm). (B) Photos of the brain slice demonstrate that corticostriatal inputs to SPNs were tested using bipolar electrodes placed over the corpus callosum (left), while thalamo-cortical inputs to SPNs were tested by placing the bipolar electrodes at the reticular nucleus (right; Bar: 1 mm). (C) Photos obtained during electrophysiology recordings show dye-filled SPNs without (left) and in the presence of channel rhodopsin (right; Bar: 50 µm).
